# *Lactococcus lactis* Strain Plasma Intake Suppresses the Incidence of Dengue Fever-like Symptoms in Healthy Malaysians: A Randomized, Double-Blind, Placebo-Controlled Trial

**DOI:** 10.3390/nu13124507

**Published:** 2021-12-16

**Authors:** Chee-Sieng Khor, Ryohei Tsuji, Hai-Yen Lee, Siti-Sarah Nor’e, Norhidayu Sahimin, Adzzie-Shazleen Azman, Vunjia Tiong, Pouya Hasandarvish, Boon-Teong Teoh, Yih-Harng Soh, Jian-Hai Chai, Takeshi Kokubo, Osamu Kanauchi, Naoki Yamamoto, Sazaly AbuBakar

**Affiliations:** 1 Tropical Infectious Diseases Research & Education Centre (TIDREC), Universiti Malaya, Kuala Lumpur 50603, Malaysia; khor_cs@um.edu.my (C.-S.K.); leehaiyen@um.edu.my (H.-Y.L.); sitisarahnore@um.edu.my (S.-S.N.); ayusahimin@um.edu.my (N.S.); adzzieshazleen.azman@monash.edu (A.-S.A.); evationg@um.edu.my (V.T.); pouyahassandarvish@um.edu.my (P.H.); boonteong@um.edu.my (B.-T.T.); snoopy84@gmail.com (Y.-H.S.); leo_2086@yahoo.com (J.-H.C.); 2 Kirin Central Research Institute, Kirin Holdings Co., Ltd., Yokohama 236-0004, Japan; Ryohei_Tsuji@kirin.co.jp (R.T.); takeshi_Kokubo@kirin.co.jp (T.K.); 3 Research and Development Strategy Department, Kirin Holdings Co., Ltd., Tokyo 164-0001, Japan; kanauchio@wonderfarmonline.com; 4 Genome Medical Sciences Project, National Center for Global Health and Medicine, Chiba 272-8516, Japan; nyamamoto0508@gmail.com; 5 Tokyo Medical and Dental University, Tokyo 113-8519, Japan

**Keywords:** dengue virus, dengue fever, LC-Plasma, *Lactococcus lactis*, lactic acid bacteria

## Abstract

Dengue fever (DF) is a mosquito-borne disease still with no effective treatment or vaccine available. A randomized, placebo-controlled, double-blinded, parallel-group trial was undertaken to evaluate the efficacy of oral intake of *Lactococcus lactis* strain plasma (LC-Plasma) on the presentation and severity of DF-like symptoms among healthy volunteers. Study participants (320) were assigned into two groups, and consumed either placebo or LC-Plasma tablets (approximately 100 billion cells/day) for 8 weeks. The clinical symptoms of DF were self-recorded through questionnaires, and exposure to DENV was determined by serum antibody and/or DENV antigen tests. No significant differences between groups were observed for exposure to DENV, or the symptomatic ratio. Results obtained showed that participants from the LC-Plasma group reported a significant reduction in the cumulative incidence days of DF-like symptoms, which include fever (*p* < 0.001), muscle pain (*p* < 0.005), joint pain (*p* < 0.001), and pain behind the eyes (*p* < 0.001), compared to that of the placebo group. Subgroup analysis revealed a significantly (*p* < 0.05) reduced severity score in the LC-Plasma group when study sites were separately analyzed. Overall, our findings suggest that LC-Plasma supplementation reduces the cumulative days with DF-like symptoms, and the severity of the symptoms. Daily oral intake of LC-Plasma, hence, is shown to mitigate the DF-like symptoms.

## 1. Introduction

DF is one of the most common vector-borne diseases worldwide. The incidence of DF occurs mostly in tropical and subtropical regions of the world. Dengue virus (DENV), the causative agent of DF, is transmitted to humans during the blood meal of the DENV-infected female *Aedes* mosquitoes. Infected individuals may appear asymptomatic [1], or present with mild undifferentiated illnesses which can develop into severe forms of the disease with pronounced intravascular leakages, and multiple organ dysfunction [2,3,4]. If left untreated, the disease in its severe forms could lead to death. It has been estimated that 390 million DF cases occur globally each year, with 294 (75.4%) million of the cases being inapparent [5]. There is no effective medication for the treatment of DF, and the only approved prophylaxis is Dengvaxia^®^ (Sanofi Pasteur, Swiftwater, PA, USA), a live recombinant tetravalent vaccine with a vaccine efficacy at approximately 65.6% (95% CI, 60.7 to 69.9) [6].

Malaysia, a tropical country with DF reported throughout the year, reported annual cases of more than 80,000 in recent years, with the incidence peaking during the monsoon season [7]. The high DF incidence resulted in estimated annual medical-related expenses for DENV-related illness of at least USD 102 million each year [8]. The economic burden caused by DF is estimated to be much higher in comparison to the medical expenses, as it also includes costs incurred for surveillance, research, and vector prevention and control activities [8,9,10]. Indirectly, the infection results in reduced workdays, which contributes to further economic losses to the individuals and nation [8,11]. The absence of effective methods to mitigate the spread of dengue, and measures to effectively treat DF, especially in dengue-endemic regions, are among the reasons that novel approaches that are effective, yet easy to implement, are urgently needed.

Studies have shown that the innate immune system plays an important role as the first line of defense against infections, through recognition and activation of antiviral molecules, such as interferons (IFNs), interferon-stimulated genes (ISGs), and inflammatory cytokines [12]. The role of detecting and orchestrating the response is played by the dendritic cells, under which, the plasmacytoid dendritic cells (pDC) are the most well known for their role as an antiviral response. The pDC can stimulate a high amount of production of type I IFN, 1000-times more than other types of cells [13].

The pDC can be stimulated in vitro and in vivo by the lactic acid bacteria (LAB). LAB is a large group of rod- or cocci-shaped gram-positive bacteria commonly used in the production of food and supplements with probiotics. Ingestion of LAB can aid in lactose digestion, help to prevent and treat diarrheal diseases, as well as enhance the immune system of the digestive tract [14,15]. *Lactococcus lactis* strain plasma (herein forth, LC-Plasma) is a synonym of *Lactococcus lactis* subsp. *lactis* JCM 5805, which showed unique functions to directly activate pDCs, which, in turn, upregulate the production of type I and III IFNs via TLR9 stimulation [16].

An earlier community-based study suggested that supplementation of the diet with regular oral administration of LC-Plasma confers protection against influenza-like illness through upregulation of the IFN-α mediated responses [17]. Apart from eliciting protection against influenza-like illness, IFN-mediated responses are also essential in the clearance of DENV, and inhibition of its infection and replication [18,19]. The humoral factors, induced by mouse bone-marrow-derived DCs via LC-Plasma stimulation, clearly suppressed the DENVs replication in vitro [20], and oral administration of LC-Plasma showed faster clearance of DENVs i.p. infection in vivo [21]. Hence, it is postulated that the consumption of LC-Plasma-containing products may confer protection against DENV infection and DF-like illnesses in humans, similarly through upregulation of IFN production. Here, a randomized, double-blinded, placebo-controlled trial is undertaken to determine the efficacy of LC-Plasma oral supplementation in preventing DENV infection, and reducing the incidence of dengue fever-like symptoms among healthy Malaysian participants.

## 2. Materials and Methods

### 2.1. Study Design and Participants

The protocol of this trial and all related amendments were reviewed and approved by the Universiti Malaya Medical Centre Medical Research Ethics Committee (Trial protocol number: 20161219-4683), and they were subsequently reviewed by the Clinical Research Ethics Committee of Kirin Holdings Co., Ltd, Tokyo, Japan. (Trial protocol number: 2017-003). This trial was conducted following the International Code of Harmonization Good Clinical Practices (ICH-GCP), and the principles under the Declaration of Helsinki, and the protocol was registered in the University Hospital Medical Information Network (UMIN) Clinical Trials Registry (No. UMIN000038386)

This trial was a randomized, placebo-controlled, double-blinded group trial in healthy Malaysian volunteers residing in the selected sites. Participants were given the study products to be consumed for a continuous 16 weeks. This study was conducted from September 2019 to July 2020. The study was divided into four sequential phases: Phase 1 to Phase 4.

#### 2.1.1. Phase 1: Enrollment, Screening/Eligibility Phase

In Phase 1, we selected the trial population, which consisted of adult Malaysian citizens aged 18 years old and older, residing in the Ministry of Health (MOH) Malaysia identified persistent DF hotspot areas, Site 1, known as Desa Mentari (DM); and Site 2, Mentari Court (MC), two high-density residential areas in the locality of Petaling Jaya district, Selangor [22]. The number of participants targeted in this trial was set at 320 participants. The rationale of this number of participants was based on the calculation of 5.5% precision, and a 95% confidence interval of an estimated 2923 local adults living in the two trial sites. The minimum sample size required was 286, and it was estimated that 10% of the recruited participants will drop out due to non-compliance, developing underlying diseases, or pregnancy. After including the dropout rate factor, an estimated number of 320 participants were targeted. A total of 510 healthy adults were screened at the trial sites. The participants were informed of the trial procedure; and the fulfillment of inclusion and exclusion criteria (listed in Table S1) with the physical examination, questionnaire, and laboratory screening through blood biochemical profile and anti-DENV IgM ELISA, and decided voluntarily to participate in this intervention with a signature on the informed consent form. Information from the enrollment was screened, and a total of 320 participants were selected to participate in the trial. This phase was conducted from 7 September 2019 to 20 October 2019.

#### 2.1.2. Phase 2: The Randomization and Baseline Visit

**Figure 1:**
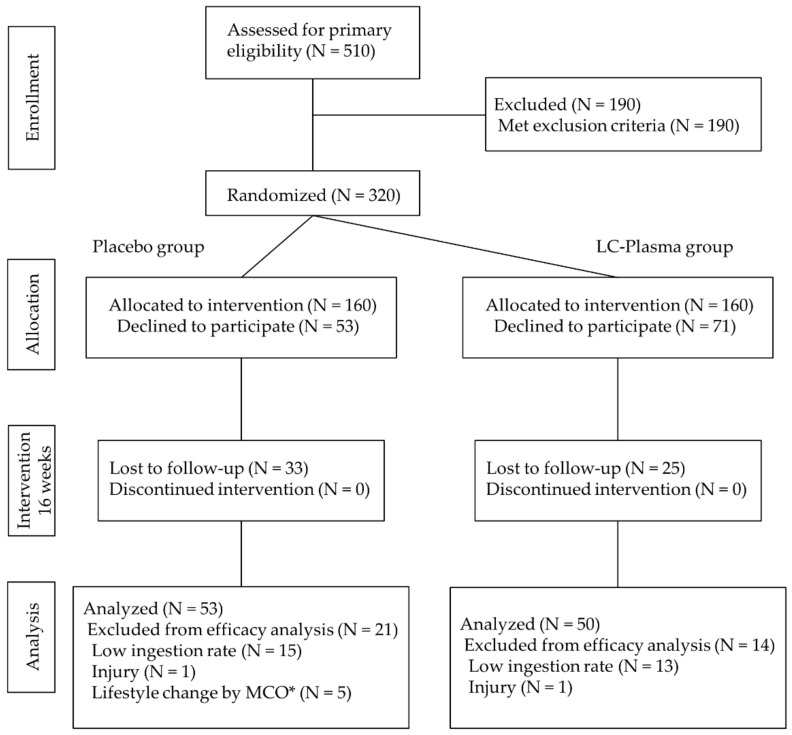
Flow chart diagram of this trial. * MCO, movement control order, implemented by the Malaysian government due to the COVID-19 pandemic.

In Phase 2, qualified participants identified through the preliminary screening were randomized into two groups (placebo group and LC-Plasma group) by employing stratified randomization (XLSTAT, Addinsoft, Paris, France) with distribution of age, gender, trial sites, residential location (including residential unit blocks and floors), BMI, anti-DENV IgG level, and routine mosquito control activities being taken into consideration (Figure 1). Randomization was performed by clinical investigators. Document controller, a professor from Universiti Malaya who was not related to the study, assigned participants to the interventions. Investigators, participants, and document controllers were blinded to the assignments of interventions throughout the study.

Qualified participants (hereinafter, participants) were notified and invited to attend the scheduled baseline visit. During the site visit, participants were required to undergo a basic physical examination and donate blood samples for baseline data points. Participants were given a self-assessment (SA) booklet, assigned the study product (placebo and LC-Plasma), and an explanation of their responsibilities over the trial period. Participants were required to consume the study product daily, and record their daily health conditions in the SA booklet. The baseline trial visit was performed from 14 December 2019 to 22 December 2019.

#### 2.1.3. Phase 3: The Follow-Up Visit

In Phase 3, site visits were conducted approximately 8 weeks after the baseline trial visit (from 15 February 2020 to 23 February 2020). Participants returned the used study product container, completed the SA booklet, and took a physical examination and blood sampling. After that, they were given a new SA booklet, and assigned the study product similar to that received in Phase 2.

#### 2.1.4. Phase 4: Final Visit

In Phase 4, at 16 weeks after the baseline visit (from 4 April 2020 to 12 April 2020), participants were to return the used study product container, and complete the SA booklet, take a physical examination, and do blood sampling.

### 2.2. Self-Assessment (SA) Booklet

Participants were each given a SA booklet to record their daily intake of the study product, as well as monitoring any symptoms associated with DF- or influenza-like illnesses (listed in the SA booklet), bowel movements, diet restrictions (no fermented foods), intake of any other medications, and hospitalization. In the SA booklet, self-assessment on the severity of any symptoms was classified using a 5-scale degree of severity (1—none, 2—slight, 3—moderate, 4—severe, 5—very severe). Assessment of symptoms included fever, headache, muscle pain, joint pain, pain behind the eyes, sore throat, cough, runny nose, sneezing, vomiting, and diarrhea. Additional columns were provided for any other symptoms not listed.

### 2.3. Study Products

The study products were formulated based on the previous human clinical trial [23] whereby a dose of approximately 1.0 × 10^11^ cells of LC-Plasma was able to elicit pDC activation, and improvement of immune responses. Therefore, the constituent of the intervention product was determined to be at 1.0 × 10^11^ cells, which were equivalent to two tablets per day, and the placebo product was set to contain the same volume of maltodextrin instead of LC-Plasma, so as not to identify between the two products. The study products were manufactured under GMP conditions following approval of the Malaysia National Pharmaceutical Regulatory Agency (NPRA), and the Ministry of Health Malaysia under the Clinical Trial Exemption License (CTX-190402). The participants were given sufficient study products for a period of 8 weeks in each dispensing during the site visit.

### 2.4. Blood Collection and Analysis

Medical assistants and certified phlebotomists performed blood withdrawal by venipuncture. The blood samples were used to perform complete blood count with differential, lipid profile testing, liver function test, and blood electrolytes test. In addition, levels of full blood count, including liver function enzymes, creatine phosphokinase (CPK), urea nitrogen, creatinine, uric acid, calcium (Ca), inorganic phosphorus (P), magnesium (Mg), serum iron (Fe), blood glucose, and HbA1c, were measured, as well as for safety data analysis. The blood samples were also tested for hepatitis B virus, hepatitis C virus, human immunodeficiency virus, and human T-lymphotropic virus type 1.

### 2.5. Anti-DENV Antibodies (IgM & IgG) and Antigen (NS1) Screening

The presence of anti-DENV antibodies and antigens were screened using the Standard F Dengue IgM/IgG FIA kit (SD Biosensor, Suwon, Korea), and Standard F Dengue NS1 Ag kit (SD Biosensor, Suwon, Korea) [24,25]. The assays were performed strictly following the manufacturer’s provided instructions. Sera tested for anti-DENV antibodies were considered positive if the readings obtained were above the cut-off index of 1.0 (COI > 1.0), whereas samples tested for DENV NS1 antigen were considered positive when the readings obtained were above 0.5 (COI > 0.5).

### 2.6. Anti-SARS-CoV-2 Antibodies Screening

The emergence of the COVID-19 pandemic during the trial necessitated detection for the presence of anti-SARS-CoV-2 IgM and IgG antibodies. The antibodies were screened using the anti-SARS-CoV-2 NCP ELISA (IgM) kit (Euroimmun, Lubeck, Germany), and anti-SARS-CoV-2 ELISA (IgG) kit (Euroimmun, Lubeck, Germany), respectively. The assays were performed strictly following the instructions provided by the kit manufacturer. Sera tested were considered positive if the optical density (OD) readings obtained were 1.1 times or higher than OD readings from the calibrator [26,27].

### 2.7. DENV Quantitative Reverse Transcription Polymerase Chain Reaction (qRT-PCR)

RNA extraction was performed using the MagMAX™ Viral RNA Isolation Kit (ThermoFisher Scientific, Waltham, MA, USA), and KingFisher™ Flex Purification System (ThermoFisher Scientific, Waltham, MA, USA). The RNA extraction was performed according to the manufacturer’s specifications.

The presence of DENV RNA in the extracted samples was quantified using a qRT-PCR protocol, which has been validated under the RCPA-QAP program. A total of 2.0 μL of RNA samples was added into 13.0 μL of qRT-PCR master mix (SensiFAST™ Probe Hi-ROX One-Step Kit (Meridian Bioscience, London, UK), forward and reverse primer (Integrated DNA Technologies, Singapore), and probe (Integrated DNA Technologies, Singapore)), resulting in the final mixture of 15.0 μL for qRT-PCR. qRT-PCR was performed in a StepOnePlus Real-Time PCR system (Applied Biosystem, Foster City, CA, USA) under the following conditions: 1 cycle of 45 °C for 10 min (reverse transcription) and 95 °C for 2 min (polymerase activation), followed by 40 cycles of 95 °C for 5 seconds (denaturation) and 60 °C for 20 seconds (annealing/extension).

Results obtained were analyzed using the Applied Biosystems StepOnePlus software (Applied Biosystem, Foster City, CA, USA). Samples with a Ct-value (cycle threshold-value) of less than 40.0 were considered positive for DENV RNA.

### 2.8. Definition of Exposure to DENV

Participants enrolled in the intervention were considered as exposed to DENV if either of the following criteria was fulfilled [24,25,28]:

- Tested positive for anti-dengue IgM (COI > 1.00) with at least a 4-fold increase in OD reading as compared to the primary sample.
- Tested positive for dengue NS1 antigen (COI > 0.5) with at least a 4-fold increase in OD reading as compared to the primary sample.
- Tested positive for DENV RNA with a Ct-value of less than 40.0 (Ct < 40).

Note: COI values stated were determined by the respective kit manufacturer.

### 2.9. Statistical Analysis for Outcomes

The background information consisted of age, height, weight, blood pressure, and pulse. A Mann–Whitney U test was performed to determine if any of the parameters tested were significantly different between the placebo and LC-Plasma groups. The statistical analyses were performed using the SPSS software V26 (IBM Corporation, New York, NY, USA).

For the symptom severity score, the Chi-squared test was used to test for statistical differences of DENV-exposed ratio, symptomatic ratio, and cumulative incidence days of clinical symptoms between LC-Plasma and the placebo groups. Wilcoxon’s rank-sum test was performed to test for statistical differences of average scores of clinical symptoms throughout the intervention period between the LC-Plasma and the placebo groups. Subgroup analysis was performed to further explore the effects of LC-Plasma given the impact of residential sites. Statistical analyses were performed with R software (Version 3.6.2, R Core Team) or Excel toukei (Version 3.20, Bellcurve, Tokyo, Japan).

The safety data assessment was performed based on 44 parameters in the blood analysis. The Mann–Whitney U test was performed to compare the groups using SPSS software (Version 25, IBM Corporation, New York, NY, USA). A significant difference was defined as *p* < 0.05, and a moderate difference was defined as *p* < 0.1.

## 3. Results

### 3.1. Rescheduling of Site Visits Due to COVID-19 Pandemic Movement Control Order (MCO)

During the study period, on 11 March 2020, the World Health Organization (WHO) declared a COVID-19 pandemic caused by SARS-CoV-2. During that time, Malaysia experienced an escalating number of cases, resulting in the imposition of the MCO throughout the country on 18 March 2020, which lasted until 9 June 2020. Under the MCO, all non-critical movements were prohibited, and that included all research activities. The planned trial intervention period scheduled for 16 weeks was interrupted. The MCO was later replaced with the Recovery Movement Control Order (RMCO) from 10 June 2020. Under the RMCO guidelines, certain research activities were allowed, hence, the final study visit was rescheduled, and implemented after both IRBs approved the amended study plan. The participants were requested to attend the postponed site visit scheduled from 1 July 2020 to 26 July 2020, and they were also requested to return the used and unused study product container, and complete the SA booklet. In addition, participants were required to undergo a basic physical examination, and volunteered blood samples.

During the intervention period, the COVID-19 pandemic, and related Malaysian government countermeasures, significantly impacted the lifestyles and environment of the study participants, especially from the 9th week onward. Furthermore, the three months delay in implementing the final study visit resulted in higher chances of being exposed to DENV without a sufficient supply of the study products. As such, only data obtained before the imposition of MCO were analyzed to avoid potential sampling bias.

### 3.2. Participant Characteristics and Analysis Object

The consolidated standards of reporting trials flow diagram for the study is as shown in Figure 1. Five hundred and ten candidates were recruited, and three hundred and twenty participants were enrolled. In this trial, the intervention period was planned for 16 weeks with three site visits (start point, follow-up point (8 weeks after start of intervention), and final point). Eligible participants were randomly allocated into two groups: placebo group (N = 160), and LC-Plasma group (N = 160). Fifty-three participants in the placebo group, and seventy-one participants in the LC-Plasma group declined to participate after the allocation. Additionally, thirty-three participants in the placebo group, and twenty-five participants in the LC-Plasma group were lost to follow-up during the intervention period. Twenty-one participants in the placebo group, and fourteen participants in the LC-Plasma group were excluded from analysis due to the low ingestion ratio (<80%), persistent muscle and joint pain caused by old injuries, and drastic lifestyle changes due to the MCO. Finally, 53 participants in the placebo group, and 50 participants in the LC-Plasma group were analyzed to evaluate the efficacy of LC-Plasma supplementation. The baseline condition of analyzed subjects was shown in Table 1. The baseline characteristics of the participants in each group based on age, weight, height, blood pressure, and pulse revealed no significant differences between the groups throughout the entire trial.

Of the 103 participants whose data were included in the analysis, 44 participants were residents from DM, with the rest (N = 59) from MC. Mean age of participants from DM (43.2 ± 13.2) was significantly (*p* < 0.05, 95% CI = 5.96–16.07) higher than participants from MC (32.2 ± 12.2). Participants from both study sites were predominantly females, with 77.3% (N = 34) and 71.2% (N = 42), respectively.

### 3.3. Number of DENV Exposure and Symptomatic Ratio

During the intervention, a total of nine participants (four participants in the LC-Plasma group, and five participants in the placebo group) were considered to have been exposed to DENV (Table S2). The identification of exposure to DENV was through laboratory testing using qRT-PCR, ELISA for detection of NS1 antigen, and ELISA for anti-DENV Abs. The definition of “Exposed” to DENV was as described in Material and Methods. Additionally, according to the SA booklet, of these participants, the clinical trial physician diagnosed a total of three participants (one participant in the LC-Plasma group, and two participants in the placebo group) presented with symptomatic DF. A Chi-squared test was performed on the data to investigate the difference of DENV exposure ratio and DF symptomatic ratio between two groups, and found that there were no significant differences in both indices between the two groups (Table 2).

### 3.4. Comparison of Cumulative Symptomatic Days and Severities of Each Clinical Symptom

Throughout the intervention period, general health and clinical symptoms of the participants were recorded in the SA booklet with five grades: (1) none, (2) slight, (3) moderate, (4) severe, and (5) very severe. To investigate the effects of LC-Plasma on the onset and duration of clinical symptoms, the severity scores were divided into two groups: “without symptoms” (score 1), and “with symptoms” (sum of score 2 to 5, symptom positive despite intensity), and the cumulative symptomatic days of each clinical symptom were compared between two groups using a Chi-squared test. From the analyses, the cumulative symptomatic days of “Fever”, “Headache”, “Muscle pain”, “Joint pain”, “Pain behind eyes”, “Sore throat”, “Cough”, “Runny nose”, and “Sneezing” in the LC-Plasma groups were significantly lower than those in the placebo group (*p* < 0.05) (Table 3). The cumulative symptomatic days of “Vomit” in the LC-Plasma group were moderately lower than those in the placebo group (*p* = 0.055). The symptoms of “Fever”, “Headache”, “Muscle pain”, “Joint pain”, and “Pain behind eyes” were known as among the typical symptoms of DF [29,30,31], and those of “Fever”, “Headache”, “Sore throat”, “Cough”, and “Runny nose” were known as representative symptoms of respiratory illnesses [32].

**Figure 2:**
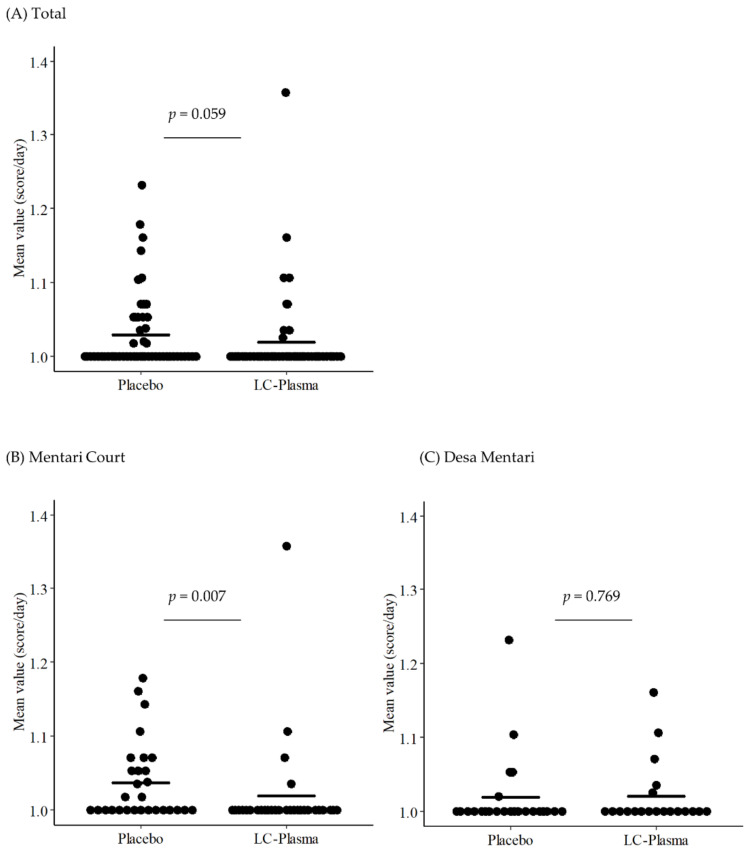
Suppression of fever scores by LC-Plasma supplementation among: (**A**) total participants, (**B**) Mentari Court participants, and (**C**) Desa Mentari participants.

The effects of LC-Plasma supplementation on the severity of clinical symptoms were calculated using the mean score value (score/day) of each participant during the intervention period, then the scores between two groups were compared using the Wilcoxon’s rank-sum test. The analyses showed that the score of fever in the LC-Plasma group (mean ± SD = 1.019 ± 0.059) was moderately lower (*p* = 0.059) than that of the placebo group (mean ± SD = 1.029 ± 0.052) (Figure 2A, Figures S1A and S2A and Table S3). There were no other symptom differences recorded between the two groups.

### 3.5. Subgroup Analysis for DF-like Symptoms Focusing on the Residence

The two chosen study sites were among those listed as high-density populations in Kuala Lumpur with a persistently high incidence of DF. The demographics between the sites, however, were different. Participants from MC (mean age ± SD = 32.2 ± 12.2) were significantly (*p* < 0.05) younger compared to those from DM (mean age ± SD = 43.2 ± 13.2). The age difference could exert different responses to the trial, as functions of immune cells were reported to decline with aging [33]. To investigate the possibilities, subgroup analysis based on residence was performed, but limited to only the DF-like symptoms. Results from the analyses showed a significantly lower severity score (*p* < 0.05) in fever, headache, and joint pain exhibited by MC participants (Table 4, Figure 2B, Figures S1B and S2B). However, none of the symptoms were significantly different when only DM participants were analyzed (Table 4, Figure 2C, Figures S1C and S2C).

### 3.6. Number of SARS-CoV-2 Exposed Participants

Since the intervention period of this trial overlapped with the COVID-19 pandemic period, we investigated the number of participants who could have been exposed to the SARS-CoV-2 infection as a post-hoc test by determining the presence of anti-SARS-CoV-2-specific IgM and IgG in study participants’ sera. No participants with antibodies specific to SARS-CoV-2 were found (data not shown).

### 3.7. Safety Evaluation

Analyses of the overall biochemical blood profiles revealed no significant differences between the groups at baseline and the end of the intervention. Few of the parameters exceeded the normal range; however, no significant differences were observed between the two groups (data not shown). Throughout the trial period, three potential adverse events were reported. All three events were deemed not related to the consumption of LC-Plasma by the clinical trial physician. Hence, clinical trial physicians concluded that there were no significant adverse effects related to LC-Plasma administration in this trial.

## 4. Discussion

As previously reported, LC-Plasma has been shown to induce a high level of IFNs production from pDCs in vitro and in vivo [34,35]. The high level of IFN affected DENV infection in a mouse model [21]. Furthermore, several studies in human DENV infection have suggested the importance of early IFN responses in mitigating the effects of the infection [36,37,38]. No studies, however, have shown that intake of LC-Plasma would affect the outcome of DENV infection or DF incidence in humans. In the present study, the effects of consuming LC-Plasma on the symptoms of DF were investigated and compared to that of the symptoms of common respiratory illnesses.

There have been only limited reports on human trials investigating the impact of consuming lactic acid bacteria on viral infection, including DENV infection. Use of the LC-Plasma, however, was shown to promote the IFN production in vitro, as well as in humans [16,21]. pDCs mediate antiviral activities through interactions with the NK cells, B cells, and T cells [34,39,40]. The pDCs play a key role in innate antiviral immunity through the production of a high amount of IFN-α. IFN response to DENV infection is important in limiting the infection [36,37], possibly through triggering TLR7 after cell-to-cell contact with a member of the flavivirus [41]. IFN has been shown to upregulate the C19orf66 gene, which suppresses DENV replication in an in vitro study [18]. In addition, in an earlier study, decreased production of type I IFN by pDC was suggested to contribute to severe DF [41].

Regular consumption of LC-Plasma was previously reported to significantly decrease symptoms (sore throat and lassitude) associated with the common cold and influenza-like illnesses [23,42]. Findings from the present trial further support these findings, and, in addition, provide evidence that consumption of LC-Plasma also ameliorated the symptoms typically associated with DF. This finding is consistent with the previous intervention study, in which the overall cumulative incidence rate was two-thirds lower among the children who consumed the LC-Plasma-containing yogurt of the same active ingredient in this study [42]. The previous trial reported the anti-viral immunity among healthy adults in Japan after consumption of LC-Plasma reduced the incidence rate of common cold and influenza [23]. The protective effects of LC-Plasma activation of pDC, which in turn, induced IFN-α, however, have been widely observed in multiple earlier studies on viral infections [17,23,42,43].

In the study, though both MC and DM were sites previously identified with persistently high DF incidence, the demographics of the populations were quite different. For DM, the population was from the Hardcore Poor Housing (HPH) squatters’ resettlement scheme, mainly comprising of people with Malay and Indian ethnicities [44]. Whereas in MC, it is a low-cost housing area inhabited by the different ethnicities of Malay, Chinese, and Indian and migrant workers [45]. Participants for this clinical trial in the two study sites, however, are mainly Malay and Indian ethnicities. Whether their genetic backgrounds would influence the trial outcome would require further investigation in the future.

Through subgroup analysis of study sites, however, contrasting findings between MC and DM were revealed. The severity scores of “fever”, “headache”, and “joint pain”, which were not significantly different without subdivision, were found to be significantly different when data obtained from DM were excluded (*p <* 0.05). The symptom scores of the placebo group of participants from MC were generally higher than the placebo group in DM. Hence, the ameliorating effects of LC-Plasma consumption among the symptomatic participants from MC became significant. We currently do not know if the lower symptom scores recorded by the participants from DM correlate with the demographic presentation of the population. It was earlier noted that between the two populations, the biochemical profiles resulted in higher exclusions of those residing in DM (50.4%) in comparison to MC (21.6%) during the initial screening of the participants. This raises the possibility that the population residing in DM, in general, has a higher tolerance of the symptoms, resulting in lower reporting of severity symptom scores.

During the study period (January to February 2020), a total of 14,483 dengue cases from the state of Selangor were recorded, fifty-eight of which were from the study sites [46,47,48,49,50,51,52]. The estimated population size of the state was 6.58 million (Selangor), and 30,000 reside in MC and DM. Hence, the incidence rate of DF in Selangor, and study sites during the two months were 0.22% and 0.19%, respectively. These reported figures were much lower than the actual DENV detection rate found in this study, where 8.74% (N = 9/103) of the study subjects tested positive for DENV exposure. Five of the cases were from the placebo group, whereas four were from the LC-Plasma group. The findings were not surprising, however, considering that DM and MC have persistently been listed as DF hotspots [22]. Furthermore, since the official reporting of dengue cases relies on laboratory confirmation among those who sought medical treatment in healthcare facilities, those with asymptomatic or mild infections are otherwise not recorded. In contrast, in the present trial, all study participants, regardless of their clinical presentations, were laboratory-tested. The findings, hence, highlighted the high incidence of unreported dengue infections in the dengue hotspot areas [53].

In the study, participants were assessed based on health questionnaires, and their blood profile was analyzed at the start of the intervention period, and after 8 weeks. The 8-weeks post-intervention data obtained from this study did not detect any significant differences (*p* > 0.05) in blood profiles between the placebo and LC-Plasma groups. There were also no adverse events attributable to the consumption of LC-Plasma throughout the study. These findings are consistent, and conform to the observation that LC-Plasma has been marketed as iMUSE™ for 5 years without report of any detrimental effects [54,55]. Similarly, no adverse events were associated with LC-Plasma in all other clinical trials conducted [23,42,43,56]. This evidence contributed further to the safety profile of LC-Plasma.

In this study, DM and MC were chosen as the trial sites since both sites have persistently recorded high incidences of DF over the last 4 years [22]. However, the emergence of the COVID-19 pandemic in Malaysia during the trial period, and the subsequent implementation of the MCO impacted the conduct of the full trial, which resulted in only a minimum number of participants completing the trial. This severely reduced the statistical power required to accurately detect differences between the groups on the severity of symptoms, and ratio of exposure to symptoms of DF. A larger-scale clinical trial has to be conducted post-COVID-19 pandemic to ascertain the effects of LC-Plasma on incidences of DF. To date, there has been no clinical study reported on the potential benefit of the consumption of lactic acid bacteria against the incidences of DF. Here, it is demonstrated that eight weeks of consumption of the LC-Plasma significantly ameliorated the typical symptoms associated with DF, and reduced cumulative incidences of the symptoms. Hence, daily consumption of LC-Plasma should be considered among the approaches for the amelioration of the symptoms of DF in DENV high prevalence localities in dengue-endemic regions.

## Figures and Tables

**Table 1:** Background data of participants.

		Placebo	LC-Plasma	
Number of participants		MC: 29, DM: 24	MC: 30, DM: 20	
Gender		Male: 16, Female: 37	Male: 11, Female: 39	
	(Unit)			*p*-value
Age	year	37.69 ± 14.33	36.12 ± 13.10	0.673
Height	cm	157.72 ± 8.15	155.64 ± 9.68	0.179
Weight	kg	71.28 ± 16.67	68.11 ± 17.23	0.150
BP-Diastole	mmHg	125.54 ± 13.69	125.81 ± 12.40	0.983
BP-Systole	mmHg	79.36 ± 7.99	78.38 ± 9.25	0.414
Pulse	bpm	82.90 ± 13.09	81.74 ± 12.08	0.431

Data are shown as mean ± SD. MC: Mentari Court, DM: Desa Mentari. BP: blood pressure.

**Table 2:** Exposure rate to DENV between groups.

	Exposed	Not Exposed	*p*-Value ^1^	Symptomatic	Asymptomatic	*p*-Value ^1^
Placebo	5	48	0.797 ^2^	2	3	0.635 ^2^
LC-Plasma	4	46		1	3	

^1^*p*-value for Chi-squared test. ^2^ There were no significant differences between the two groups in dengue virus exposure ratio, and the symptomatic ratio of dengue fever.

**Table 3:** Cumulative symptomatic days.

Symptoms		Cumulative Days		
		without Symptoms	with Symptoms	*p*-Value
Fever	Placebo	2837	64	0.001 **
	LC-Plasma	2678	28	
Headache	Placebo	2706	195	0.000 **
	LC-Plasma	2633	72	
Muscle pain	Placebo	2792	109	0.005 **
	LC-Plasma	2640	66	
Joint pain	Placebo	2770	131	0.000 **
	LC-Plasma	2645	61	
Pain behind eyes	Placebo	2853	48	0.000 **
	LC-Plasma	2691	15	
Sore throat	Placebo	2761	140	0.000 **
	LC-Plasma	2643	63	
Cough	Placebo	2746	154	0.028 *
	LC-Plasma	2596	110	
Runny nose	Placebo	2711	190	0.000 **
	LC-Plasma	2628	78	
Sneezing	Placebo	2738	162	0.008 **
	LC-Plasma	2596	110	
Vomit	Placebo	2887	14	0.055 +
	LC-Plasma	2701	5	
Diarrhea	Placebo	2867	34	0.133
	LC-Plasma	2685	21	

Chi-squared test, **: *p* < 0.01, *: *p* < 0.05, +: *p* < 0.1.

**Table 4:** The severity of dengue fever-like symptoms (subgroup analysis).

Mentari Court			
	Placebo Group	LC-Plasma Group	*p*-Value
Fever	1.037 ± 0.052	1.019 ± 0.068	0.007 **
Headache	1.108 ± 0.158	1.034 ± 0.076	0.027 *
Muscle pain	1.038 ± 0.105	1.016 ± 0.053	0.218
Joint pain	1.050 ± 0.140	1.013 ± 0.050	0.028 *
Pain behind eyes	1.022 ± 0.071	1.007 ± 0.028	0.146
**Desa Mentari**			
Fever	1.019 ± 0.052	1.020 ± 0.044	0.769
Headache	1.044 ± 0.110	1.046 ± 0.086	0.535
Muscle pain	1.056 ± 0.207	1.070 ± 0.291	0.894
Joint pain	1.064 ± 0.214	1.069 ± 0.258	0.595
Pain behind eyes	1.016 ± 0.057	1.008 ± 0.029	1.000

Data are shown as mean ± SD. **: *p* < 0.01, *: *p* < 0.05.

## Data Availability

The data that support the findings of this study are not publicly available due to ethical concerns.

## Supplementary Materials

The following are available online at https://www.mdpi.com/article/10.3390/nu13124507/s1, Figure S1: Change in joint pain scores after 8 weeks intervention. The mean value of joint pain scores in each participant during the intervention period was calculated from the SA booklet, then compared between the placebo group and the LC-Plasma group. (A) The data of total participants. (B) The data of participants in Mentari Court (MC). (C) The data of participants in Desa Mentari (DM). The short line in all figures represents the mean value. Wilcoxon’s rank-sum test was performed. *p* values less than 0.05 were defined as significantly different, and *p* values less than 0.1 were defined as moderately different. Figure S2: Change in headache scores after 8 weeks intervention. The mean value of headache scores in each participant during the intervention period was calculated from the SA booklet, then compared between the placebo group and the LC-Plasma group. (A) The data of total participants. (B) The data of participants in Mentari Court (MC). (C) The data of participants in Desa Mentari (DM). The short line in all figures represents the mean value. Wilcoxon’s rank-sum test was performed. *P* values less than 0.05 were defined as significantly different, and *p* values less than 0.1 were defined as moderately different. Table S1: Enrollment selection criteria. Table S2: List of participants exposed to Dengue virus. Criteria to judge "exposed" were described as followings; IgM and/or IgG; OD was over 1.0 and elevated more than 4 times from previous blood analysis, NS1; OD was over 0.5 in blood of follow-up visit or final visit, qPCR; Ct-value is less than 40. Table S3: Severity of all clinical symptoms (total analysis). Data are shown as Mean ± SD. Wilcoxon’s Rank Sum Test was conducted, +: *p* < 0.1. The average scores were calculated according to the scores filled by each participant in their self-assessment booklet until (former) 8 weeks of intervention.
