# Supplementary material for: Lactococcus lactis Strain Plasma Intake Suppresses the Incidence of Dengue Fever-like Symptoms in Healthy Malaysians: A Randomized, Double-Blind, Placebo-Controlled Trial
Source: Nutrients. 2021 Dec 16;13(12):4507. doi: 10.3390/nu13124507 (PMC8707015; doi:10.3390/nu13124507)
Supplement: Supplementary file 1 [file nutrients-13-04507-s001.zip › nutrients-1433796-supplementary.pdf]

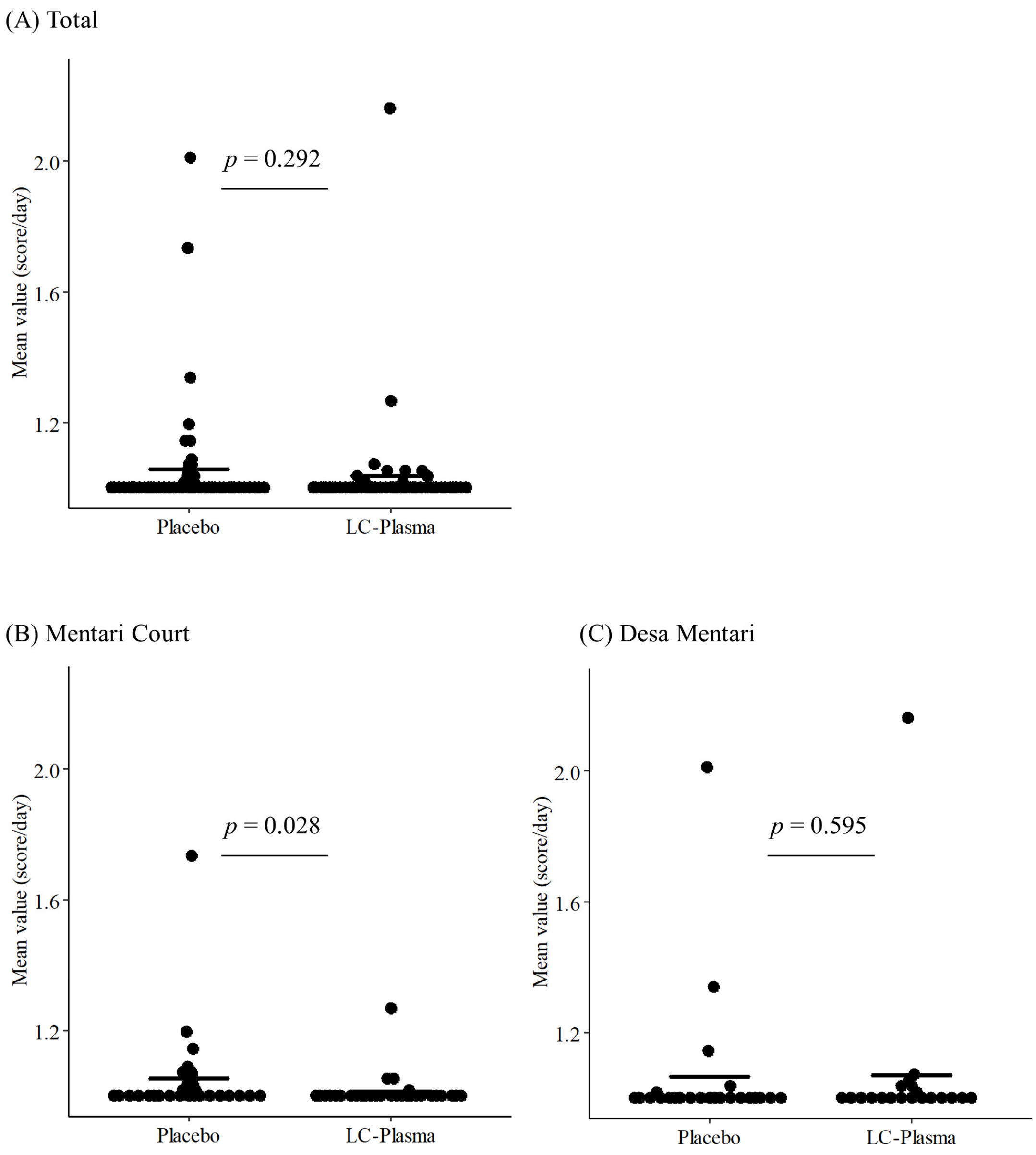

Figure S1. Change in joint pain scores after 8 weeks intervention. The mean value of joint pain scores in each participant during the intervention period was calculated from the SA booklet, then compared between the placebo group and the LC-Plasma group. (A) The data of total participants. (B) The data of participants in Mentari Court (MC). (C) The data of participants in Desa Mentari (DM). The short line in all figures represents the mean value. Wilcoxon's rank-sum test was performed. p values less than 0.05 were defined as significantly different, and p values less than 0.1 were defined as moderately different.

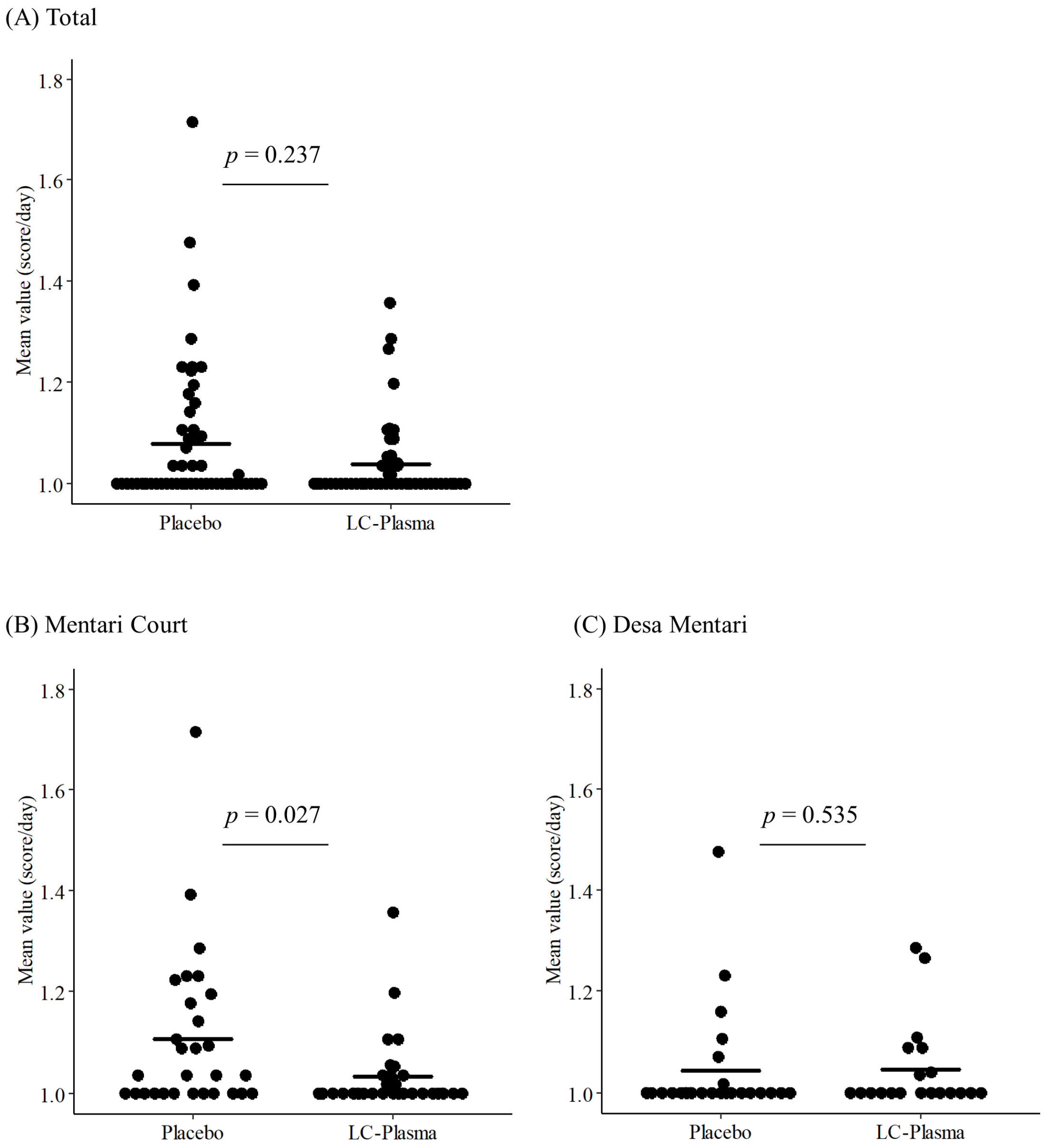

Figure S2. Change in headache scores after 8 weeks intervention. The mean value of headache scores in each participant during the intervention period was calculated from the SA booklet, then compared between the placebo group and the LC-Plasma group. (A) The data of total participants. (B) The data of participants in Mentari Court (MC). (C) The data of participants in Desa Mentari (DM). The short line in all figures represents the mean value. Wilcoxon's rank-sum test was performed. *P* values less than 0.05 were defined as significantly different, and *p* values less than 0.1 were defined as moderately different.
