# Supplementary material for: Lactococcus lactis Strain Plasma Intake Suppresses the Incidence of Dengue Fever-like Symptoms in Healthy Malaysians: A Randomized, Double-Blind, Placebo-Controlled Trial
Source: Nutrients. 2021 Dec 16;13(12):4507. doi: 10.3390/nu13124507 (PMC8707015; doi:10.3390/nu13124507)
Supplement: Supplementary file 1 [file nutrients-13-04507-s001.zip › Table S1.pdf]

Table S1. Enrollment selection criteria.

Participant Criteria	
Inclusion	Exclusion
Provide signed and dated informed consent form	Unable to provide signed and dated consent form
Willingness to comply with all study procedures for the duration of study	Unable to comply with all the study procedures
Malaysian citizen	Foreign citizen
Age of 18 years and above	Below the age of 18 years
Not feeling sick with fever for the past 2 weeks	Allergic to fermented food products
No underlying disease or sickness	Take steroids regularly
Individuals tested negative for anti-DENV IgM in the preliminary blood test	Consume foods, drinks or supplements containing probiotic on regular basis (weekly)
	Pregnant women, or intention to conceive during the study period and breastfeeding mothers
	Planned to go overseas for more than two weeks, or more than one week consecutively in total of study period
	Participating in other trials of food and pharmaceutical products
	History of digestive diseases or surgery affecting the digestive tract
	Positive for specific anti-viral antibodies/antigen (HBC, HCV,HIV or HTLV-1) in the preliminary blood screening
	Consumed alcohol within two days of blood draw
	Individuals with blood biochemistry results that is determined to be inappropriate for the study
	Individuals whom Principal Investigator or sub-investigator deems inappropriate
