# Supplementary material for: Lactococcus lactis Strain Plasma Intake Suppresses the Incidence of Dengue Fever-like Symptoms in Healthy Malaysians: A Randomized, Double-Blind, Placebo-Controlled Trial
Source: Nutrients. 2021 Dec 16;13(12):4507. doi: 10.3390/nu13124507 (PMC8707015; doi:10.3390/nu13124507)
Supplement: Supplementary file 1 [file nutrients-13-04507-s001.zip › Table S2.pdf]

Table S2. List of participants exposed to Dengue virus.

Dengue positive Participant	Definition by criteria	Group	Residence	Symptom
1	IgM & IgG	Placebo	DM	Yes
2	qPCR	Placebo	MC	Yes
3	qPCR	Placebo	DM	No
4	IgG	Placebo	DM	No
5	qPCR	Placebo	MC	No
6	IgM & IgG	LC-Plasma	MC	Yes
7	IgM	LC-Plasma	MC	No
8	qPCR	LC-Plasma	MC	No
9	qPCR	LC-Plasma	MC	No

Criteria to judge "exposed" were described as followings;

IgM and/or IgG; OD was over 1.0 and elevated more than 4 times from previous blood analysis

NS1; OD was over 0.5 in blood of follow-up visit or final visit

qPCR; Ct-value is less than 40
