# Supplementary material for: Lactococcus lactis Strain Plasma Intake Suppresses the Incidence of Dengue Fever-like Symptoms in Healthy Malaysians: A Randomized, Double-Blind, Placebo-Controlled Trial
Source: Nutrients. 2021 Dec 16;13(12):4507. doi: 10.3390/nu13124507 (PMC8707015; doi:10.3390/nu13124507)
Supplement: Supplementary file 1 [file nutrients-13-04507-s001.zip › Table S3.pdf]

Table S3. Severity of all clinical symptoms (total analysis).

Symptom	Placebo group	LC-Plasma group	*p*-value
Fever	1.029 ± 0.052	1.019 ± 0.059	0.059 +
Headache	1.079 ± 0.141	1.039 ± 0.079	0.237
Muscle pain	1.046 ± 0.158	1.038 ± 0.187	0.292
Joint pain	1.056 ± 0.176	1.035 ± 0.167	0.205
Pain behind eyes	1.019 ± 0.064	1.007 ± 0.028	0.258
Sore throat	1.070 ± 0.202	1.041 ± 0.110	0.524
Cough	1.090 ± 0.236	1.062 ± 0.172	0.848
Runny nose	1.080 ± 0.189	1.042 ± 0.105	0.856
Sneezing	1.077 ± 0.229	1.055 ± 0.162	0.933
Vomit	1.007 ± 0.024	1.003 ± 0.018	0.217
Diarrhea	1.019 ± 0.067	1.011 ± 0.047	0.441

Data are shown as Mean ± SD.

Wilcoxon's Rank Sum Test was conducted, +: $p < 0.1$

The average scores were calculated according to the scores filled by each participant in their self-assessment booklet until (former) 8 weeks of intervention.
